# Modest heterologous protection after *Plasmodium falciparum* sporozoite immunization: a double-blind randomized controlled clinical trial

**DOI:** 10.1186/s12916-017-0923-4

**Published:** 2017-09-13

**Authors:** Jona Walk, Isaie J. Reuling, Marije C. Behet, Lisette Meerstein-Kessel, Wouter Graumans, Geert-Jan van Gemert, Rianne Siebelink-Stoter, Marga van de Vegte-Bolmer, Thorsten Janssen, Karina Teelen, Johannes H. W. de Wilt, Quirijn de Mast, André J. van der Ven, Ernest Diez Benavente, Susana Campino, Taane G. Clark, Martijn A. Huynen, Cornelus C. Hermsen, Else M. Bijker, Anja Scholzen, Robert W. Sauerwein

**Affiliations:** 1 Department of Medical Microbiology, Radboud University Medical Center, Geert Grooteplein 28, Microbiology 268, 6500 HB Nijmegen, The Netherlands; 2 Radboud Institute of Molecular Life Sciences and Center for Molecular and Biomolecular Informatics, Radboud University Medical Center, Geert Grooteplein 28, CMBI 260, 6500 HB Nijmegen, The Netherlands; 3 Department of Surgery, Radboud University Medical Center, Geert Grooteplein 10, Surgery 618, 6500 HB Nijmegen, The Netherlands; 4 Department of Internal Medicine, Radboud University Medical Center, Geert Grooteplein 10, Internal Medicine 456, 6500 HB Nijmegen, The Netherlands; 5 London School of Hygiene and Tropical Medicine, Department of Pathogen Molecular Biology, Faculty of Infectious and Tropical Diseases, London, WC1E 7HT UK; 6 London School of Hygiene and Tropical Medicine, Department of Infectious Disease Epidemiology, Faculty of Infectious and Tropical Diseases, London, WC1E 7HT UK; 7 Present Address: Department of Pediatrics, Radboud university medical center, Geert Grooteplein 10, Pediatrics 804, 6500 HB Nijmegen, The Netherlands; 8 Present Address: Innatoss Laboratories B.V., Kloosterstraat 9, RE3124, 5349 AB Oss, The Netherlands

**Keywords:** *Plasmodium falciparum*, Malaria, Vaccine, Sporozoite, Controlled human malaria infection, Heterologous protection, Immune responses

## Abstract

**Background:**

A highly efficacious vaccine is needed for malaria control and eradication. Immunization with *Plasmodium falciparum* NF54 parasites under chemoprophylaxis (chemoprophylaxis and sporozoite (CPS)-immunization) induces the most efficient long-lasting protection against a homologous parasite. However, parasite genetic diversity is a major hurdle for protection against heterologous strains.

**Methods:**

We conducted a double-blind, randomized controlled trial in 39 healthy participants of NF54-CPS immunization by bites of 45 NF54-infected (n = 24 volunteers) or uninfected mosquitoes (placebo; n = 15 volunteers) against a controlled human malaria infection with the homologous NF54 or the genetically distinct NF135.C10 and NF166.C8 clones. Cellular and humoral immune assays were performed as well as genetic characterization of the parasite clones.

**Results:**

NF54-CPS immunization induced complete protection in 5/5 volunteers against NF54 challenge infection at 14 weeks post-immunization, but sterilely protected only 2/10 and 1/9 volunteers against NF135.C10 and NF166.C8 challenge infection, respectively. Post-immunization plasma showed a significantly lower capacity to block heterologous parasite development in primary human hepatocytes compared to NF54. Whole genome sequencing showed that NF135.C10 and NF166.C8 have amino acid changes in multiple antigens targeted by CPS-induced antibodies. Volunteers protected against heterologous challenge were among the stronger immune responders to in vitro parasite stimulation.

**Conclusions:**

Although highly protective against homologous parasites, NF54-CPS-induced immunity is less effective against heterologous parasite clones both in vivo and in vitro. Our data indicate that whole sporozoite-based vaccine approaches require more potent immune responses for heterologous protection.

**Trial registration:**

This trial is registered in clinicaltrials.gov, under identifier NCT02098590.

**Electronic supplementary material:**

The online version of this article (doi:10.1186/s12916-017-0923-4) contains supplementary material, which is available to authorized users.

## Background

Malaria has a significant impact on human health and economic welfare worldwide. In 2015, it caused over 200 million cases of disease and nearly half a million deaths [1]. Despite a significant decrease in malaria deaths having been observed in the last 15 years [1], the emergence of insecticide-resistant mosquitoes [2] and drug-resistant parasites [3] are threatening malaria control efforts, highlighting the need for a highly effective vaccine.

While naturally acquired immunity likely never results in sterile protection against the parasite [4], generation of long-lasting and sterilizing immunity against malaria is the goal of pre-erythrocytic vaccine approaches. So far, only one sub-unit vaccine, RTS,S (Mosquirix, Glaxo Smith Kline), has been recommended for defined clinical application [5]. This vaccine is based on a major sporozoite surface protein, the circumsporozoite protein (CSP), and has been shown to induce a short-term, 30–50% efficacy in reducing the incidence of clinical malaria in endemic areas, as well as in the controlled human malaria infection (CHMI) model [6–8]. Sterilizing immunity can be induced by attenuated whole sporozoite approaches. Immunization with radiation-attenuated sporozoites requires bites of at least 1000 infected mosquitoes to induce sterile protection in 50% of volunteers [9], or a total dose of 675 k cryopreserved sporozoites injected intravenously for full homologous protection [10]. In contrast, bites by only 30–45 malaria-infected mosquitoes [11, 12] or 150 k intravenously injected cryopreserved sporozoites [13] in the chemoprophylaxis and sporozoite (CPS) regimen induces complete sterile protection against the homologous parasite. CPS-induced protection can last for at least 2.5 years, showing unprecedented efficiency and sustainability of the protective immune response [14]. Specifically, strong effector memory T cell responses are induced [11, 12], as well as memory B cell and antibody responses targeting pre-erythrocytic stage antigens with functional activity against homologous sporozoites, inhibiting parasite development in liver cells in vitro and in vivo in human liver-chimeric mice [15–17].

Despite these advances, a major hurdle for the induction of protection against heterologous strains is the well-known genetic diversity of *Plasmodium falciparum*, allowing parasite evasion and reducing the protective efficacy. More recently, immunization with radiation-attenuated sporozoites provided 53% protection against a heterologous challenge [18]. There is also incidental evidence for CPS-induced heterologous protection in the CHMI model [19], but this has not been systematically addressed.

Here, we describe the first double-blind, placebo-controlled CHMI trial of NF54-CPS immunization by a total of 45 *P. falciparum* NF54-infected mosquitoes followed by a challenge infection with either *P. falciparum* NF135.C10 clone from Cambodia or NF166.C8 clone from Guinea. Parasites were characterized by whole genome sequencing and CPS-induced cellular and humoral responses were analyzed.

## Methods

### Study design and participants

This single center, double-blind, randomized, placebo-controlled trial was conducted at the Radboud university medical center (Nijmegen, The Netherlands) between February and November 2015. Study participants were healthy male and female volunteers (18–35 years old) with no history of malaria and screened for eligibility, including a complete medical and family history, physical examination, blood hematological and biochemical parameters, and serology for HIV, hepatitis B and C, and the asexual stages of *P. falciparum* as previously described [20]. All candidate participants provided written informed consent at the screening visit.

### Study approval

The study was approved by the Central Committee for Research Involving Human Subjects of The Netherlands (CCMO NL48732.091.14) and conducted according to the principles outlined in the Declaration of Helsinki and Good Clinical Practice standards. This trial is registered at ClinicalTrials.gov, identifier NCT02098590.

### Procedures

**Fig. 1:**
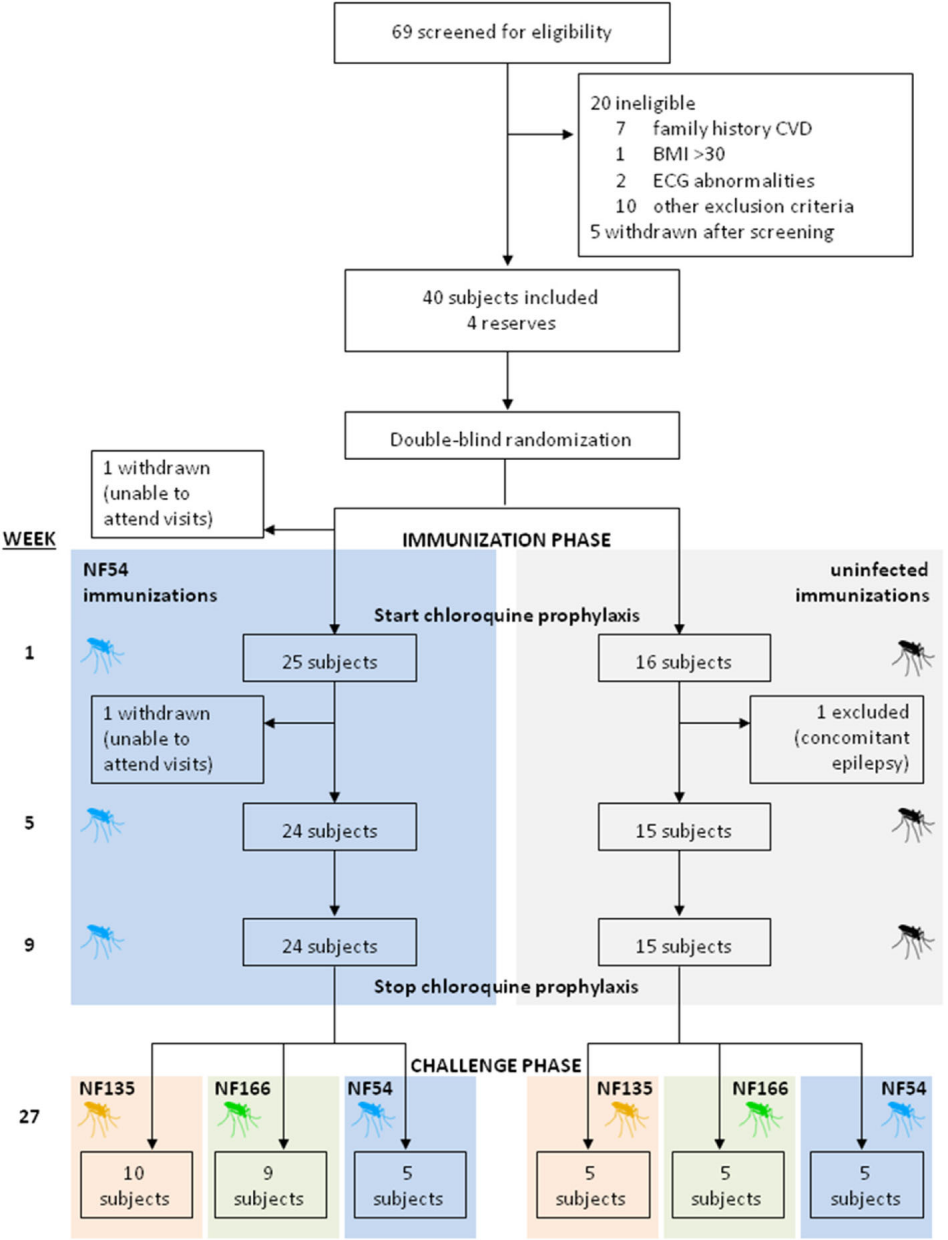
Clinical trial profile

All included study subjects (n = 41, Fig. 1) received chloroquine in a prophylactic dose (i.e., a loading dose of 300 mg of chloroquine on each of the first 2 days, followed by 300 mg once a week), for a total duration of 13 weeks. While under chloroquine prophylaxis, study groups 1, 2 and 3 received three immunizations with bites of 15 *P. falciparum* NF54-infected *Anopheles stephensi* mosquitoes. Groups 4, 5 and 6 received three mock immunizations with bites of 15 uninfected mosquitoes.

Volunteers were followed on an outpatient basis from days 6 to 10 after each immunization. Blood was examined daily, including hemocytometry, white-blood cell counts, lactic acid dehydrogenase, and highly sensitive troponin-T. From day 7 to 9, blood was also checked for malaria parasites by thick blood smear microscopy and quantitative real-time PCR (qPCR) was performed retrospectively (after study de-blinding), as described previously [21, 22].

At 14 weeks after discontinuation of chloroquine prophylaxis, all participants were exposed to bites of five *P. falciparum*-infected *A. stephensi* mosquitoes (Table 1). Subjects of groups 1 and 4 were challenged with the heterologous NF135.C10 clone, groups 2 and 5 with the heterologous NF166.C8 clone, and groups 3 and 6 with the homologous NF54 strain. Mosquitoes were examined to verify that a blood meal was taken and the presence of sporozoites in mosquito salivary glands was confirmed by dissection. If insufficient infected mosquitoes had taken a blood meal, subjects were exposed to additional mosquitoes.

**Table 1:** Baseline characteristics of subjects included in the analysis

	Group 1	Group 2	Group 3	Group 4	Group 5	Group 6
Number of participants (n = 39)	10	9	5	5	5	5
Sex						
Male	3 (30%)	2 (22%)	2 (40%)	3 (60%)	2 (40%)	5 (100%)
Female	7 (70%)	7 (78%)	3 (60%)	2 (40%)	3 (60%)	0 (0%)
Age, years	21.5 (1.8)	21.1 (2.6)	22.4 (1.6)	20.1 (1.3)	20.4 (2.5)	22.6 (3.1)
Body mass index, kg/m^2^	21.8 (2.3)	21.4 (1.7)	22.0 (3.1)	20.6 (2.0)	24.8 (3.3)	23.2 (1.7)
*P. falciparum* strain						
Immunization	NF54	NF54	NF54	Uninfected	Uninfected	Uninfected
Challenge	NF135*.*C10	NF166.C8	NF54	NF135*.*C10	NF166.C8	NF54

Data are n (%), mean (SD)

After challenge infection, subjects visited the clinical trial site twice daily from day 6 to day 15 and once daily from day 16 until day 21. Blood was drawn for parasitological assessments at every visit and safety laboratory measurements were performed once daily as described above. Malaria symptoms, including fever, headache, malaise, fatigue, myalgia, arthralgia, nausea, vomiting, chills, diarrhea, and abdominal pain, were solicited. All signs and symptoms (solicited and unsolicited) were recorded and graded by the attending physician as mild (easily tolerated), moderate (interferes with normal activity), or severe (prevents normal activity) or, in case of fever, as grade 1 (>37.5 °C to 38.0 °C), grade 2 (38.1 °C to 39.0 °C), or grade 3 (> 39.0 °C).

Subjects were treated with a curative regimen of 1000 mg atovaquone and 400 mg proguanil once daily for 3 days when parasitemia above the treatment threshold (100 parasites per milliliter of blood) was detected by qPCR [22]. Subjects that remained qPCR negative were presumptively treated with the same regimen 28 days after challenge infection. Complete cure was confirmed by two consecutive negative qPCR measurements after treatment.

### Randomization and masking

All study subjects, in two time-separated cohorts, were randomly allocated to one of the six study groups using a computer-generated list of random numbers (Microsoft Excel 2007, Redmond, WA, USA), with study groups stratified equally over each cohort. Randomization was prepared by two independent investigators and was stored securely, in sealed opaque envelopes with restricted access. Subjects, investigators and primary outcome assessors were masked to group assignment.

During the study, the homologous (NF54) challenge strain infection of the second cohort was delayed as there was no sufficiently infected batch of mosquitoes available (> 40% infected; according to our standard operating procedures). Challenge strain blinding had to be lifted for this group (six subjects), allowing them to be challenged 2 weeks later. The investigators remained blinded to immunization allocation of all participants and to the challenge strain allocation of the NF135.C10 and NF166.C8 groups until the end of the study. All study subjects and immunology assessors remained blinded during the entire study. The partial de-blinding procedure was documented and reported to the Central Committee for Research Involving Human Subjects of The Netherlands.

#### Primary study outcome

The primary outcome was the pre-patent period, namely time to parasitemia (a single qPCR measurement with a parasite density greater than 100 parasites per milliliter of blood) in subjects after challenge infection. Study sample size was determined in order to be able to detect a difference in pre-patent period of 3 days between the immunization and control groups (with α = 0.05, power = 0.90).

#### Parasites

NF54 is a longstanding and well-characterized strain isolated several decades ago from a person with airport malaria near Schiphol Airport (Amsterdam, The Netherlands) and likely originating from West Africa [23]. The NF135.C10 clone originated from a clinical isolate in Cambodia [24]. The NF166.C8 clone originated from a patient after a recent visit to Guinea (West Africa) [25].

#### Parasite culture and generation of infected mosquitoes

*P. falciparum* NF54, NF135.C10, and NF166.C8 asexual and sexual blood stages were cultured in a semi-automated culture system [26–29]. *A. stephensi* mosquitoes for immunizations and challenge infections were reared in the Radboud university medical center insectary (Nijmegen, The Netherlands) according to standard operating procedures. Infected mosquitoes were obtained by standard membrane feeding on gametocyte cultures of the different strains as described previously [29].

For in vitro sporozoite infectivity assays, salivary glands from infected *A. stephensi* mosquitoes were hand dissected and collected in complete William’s B culture medium without serum. Salivary glands were homogenized in a homemade glass grinder and the number of *P. falciparum* sporozoites was counted in a Bürker-Türk counting chamber using phase contrast microscopy [15].

#### Plasma samples

Citrated plasma samples were collected from 24 CPS-immunized volunteers at different time points using citrated vacutainer cell preparation tubes (CPT vacutainers; Becton Dickinson). Samples collected 11–14 days before the first CPS immunization (pre-immunization) and 1 day before challenge infection (18 weeks after the last immunization; post-immunization) were used for analysis of malaria antigen-specific antibody levels and assessment of functional activity in vitro. Plasma samples were stored in aliquots at – 20 °C and re-thawed no more than three times.

Prior to use for in vitro sporozoite infectivity assays in primary human hepatocytes, citrated plasma aliquots were heat-inactivated for 30 minutes at 56 °C and spun down at 13,000 rpm for 5 minutes at room temperature.

#### Peripheral blood mononuclear cell (PBMC) isolations and cryopreservation

Venous whole blood was collected in CPT vacutainers at different time points. PBMCs were isolated from peripheral blood samples, cryopreserved and stored as described previously [11]. Briefly, PBMCs were isolated by centrifugation, washed in ice-cold phosphate buffered saline, and counted in 0.1% Trypan blue with 5% Zap-o-Globin II Lytic Reagent (Beckman Coulter) to assess cell viability. Cells were cryopreserved at a concentration of 10^7^ PBMCs per milliliter in ice-cold fetal bovine serum (Gibco) containing 10% dimethylsulfoxide (Merck, Germany) using Mr. Frosty freezing containers (Nalgene). Subsequently, PBMC samples were stored in vapor-phase nitrogen. PBMC samples collected 11–14 days before the first CPS immunization (pre-immunization) and 1 day before challenge infection (18 weeks after the last immunization; post-immunization) were used for in vitro PBMC restimulation experiments and flow cytometric analysis.

#### Humoral immunological assays

##### Malaria antigen-specific antibody levels

Specific antibodies to *P. falciparum* CSP (full-length protein, obtained from Genova Biotechniques Pvt. Ltd. in Hyderabad, India [30]) in citrated anti-coagulated plasma samples were determined by a standardized enzyme-linked immunosorbent assay (ELISA) at indicated time points as previously described. Antibody levels were expressed as arbitrary units (AU) in relation to a pool of 100 sera from adults living in an area in Tanzania where malaria is highly endemic, with this positive control set at 100 AU [12, 17] (see also Additional file 1: S1 for detailed information). ELISA data analysis was performed with Auditable Data Analysis and Management System for ELISA (ADAMSEL, version 1.1) as previously described [17]. Post-immunization plasma samples were corrected for baseline responses for CSP.

##### In vitro sporozoite infectivity assay of primary human hepatocytes

To test CPS-induced functional antibody activity against sporozoite development, fresh primary human hepatocytes were isolated and cultured from patients undergoing partial hepatectomy as described in Additional file 1: S2. Briefly, viable hepatocytes (5 × 10^4^ hepatocytes/well) in complete William’s B medium were seeded into 96-well flat-bottom plates (Falcon, 353219) coated with 0.056 mg/mL rat tail collagen I per well (Roche Applied Science, 11179179-001), and cultured at 37 °C in an atmosphere of 5% CO_2_.

Two to three days after seeding of hepatocytes, batches of fresh *P. falciparum* NF54, NF135.C10, or NF166.C8 sporozoites in Williams B medium were pre-incubated with heat-inactivated naive human control serum (10% final concentration) and heat-inactivated pre- or post-CPS immunization plasma at a final concentration of 10% for 30 minutes on ice (final concentration of serum/plasma in each sample: 20%). Sporozoites pre-incubated with an anti-CSP monoclonal antibody (mAb 2A10, 10 μg/mL final concentration, MR4; MRA-183A) served as a positive control. Sporozoites in the presence of 20% heat-inactivated naive human control serum served as standard control; 5 × 10^4^ of pre-incubated sporozoites were added to 96-well plates containing monolayers of primary human hepatocytes in triplicate. Five to six days post-infection, the number of *P. falciparum*-infected hepatocytes was assessed by staining for *P. falciparum* Heat shock protein-70 and indirect immuno-fluorescence analysis using a Leica DMI6000B inverted microscope as described in Additional file 1: S3 and S4.

#### Cellular immunological assays

For the assessment of *P. falciparum*-specific cellular immune responses, pre- and post-immunization PBMCs from CPS-immunized volunteers who received NF135.C10- and NF166.C8-challenge infection were re-stimulated in vitro with cryopreserved *P. falciparum* NF54-infected erythrocytes (*Pf*RBCs), as described previously [11, 14] and in detail in Additional file 1: S5. Briefly, after thawing, PBMCs in complete culture medium (final concentration of 10 × 10^6^ cells/mL) were stimulated in vitro in duplicate with either 10^6^ cryopreserved NF54 *Pf*RBCs or 10^6^ uninfected erythrocytes (negative control) for 24 h at 37 °C with 5% CO_2_. Fluorochrome-labeled monoclonal antibody to CD107a was added for the duration of the stimulation. During the last 4 h, 10 μg/mL Brefeldin A and 2 μM monensin were added to the test wells, and 10 ng/mL PMA (Sigma-Aldrich) and 1 μg/mL ionomycin were added to positive control wells.

After 24 h of stimulation in total, cells were stained with a Live/Dead fixable dead cell stain dye and fluorochrome-labeled antibodies against the surface markers CD3, CD4, CD8, gamma delta T cell receptor, and CD56, and against the intracellular cytotoxic marker granzyme B and the cytokine interferon (IFN)-γ. Samples were kept cold and in the dark in 1% paraformaldehyde in phosphate buffered saline until measured by flow cytometry on the same day. Both time points for each volunteer were thawed, stimulated and stained within the same experimental round. Samples were acquired using a 10-colour Gallios flow cytometer (Beckman Coulter), and single stained cells were run every round for compensation. Data analysis was performed using FlowJo software (Version 10.0.8, Tree Star). Uninfected erythrocyte responses were subtracted from *Pf*RBC-specific responses for every volunteer for each time point, and post-immunization responses were corrected for pre-immunization responses.

#### Statistical analysis

Statistical analysis was performed using GraphPad Prism software (version 5, GraphPad Software Inc., California, USA). Differences in pre-patent period by qPCR between groups were determined by Log-Rank test. Differences in antibody levels across the immunization groups were analyzed with one-way ANOVA with Bonferroni’s multiple comparison post-hoc correction. For analysis of in vitro sporozoite infectivity data, differences were tested using the paired Student’s *t*-test, except when comparing between immunization groups, when a one-way ANOVA with Bonferroni’s multiple comparison post-hoc correction was used. A *P* value of < 0.05 was considered significant.

#### Genetic analysis of parasites

Genomic DNA was obtained from the three study strains and 2.3 μg of each were submitted for Illumina sequencing. The resulting fastq reads (150 bp) were aligned to the *P. falciparum* 3D7 reference genome (v.3, plasmoDB) using bowtie2 software (bowtie-bio.sourceforge.net). Single nucleotide polymorphisms (SNPs) were called with samtools and bcf/vcftools software (samtools.sourceforge.net). An alignment of all SNPs (with bcftools quality score > 100) was used to compare the study strains to others from Ghana, Guinea, Kenya, Cambodia, Thailand, and Vietnam in a combined analysis of genetic variation, as described by Campino et al. [31]. Furthermore, genes that were identified to elicit a humoral immune response in CPS immunization were examined [16]. This panel of 11 pre-erythrocytic genes including CSP and LSA-1, complemented by MAEBL, was checked for amino acid changes in the translated protein.

## Results

### Protective efficacy of CPS immunization against heterologous challenge

A total of 41 volunteers were included into the study, of whom 24 immunization and 15 placebo-control volunteers completed the clinical trial and were included in the per protocol analysis. One volunteer withdrew after being unable to attend the study visits and one was excluded due to a concomitant condition (Fig. 1). The baseline characteristics of the study population are shown in Table 1.

**Fig. 2:**
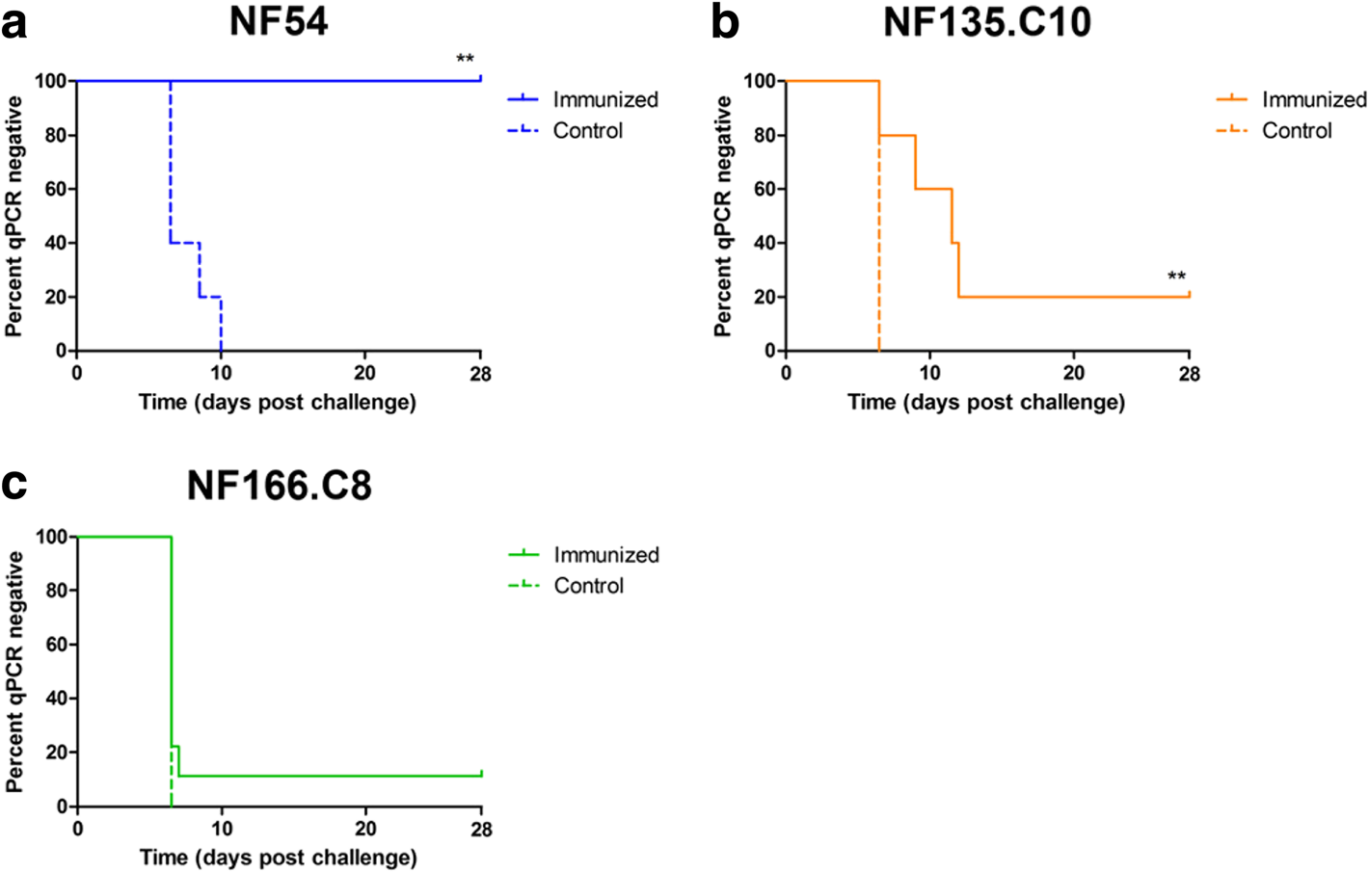
Parasitemia following homologous and heterologous challenge infection. The percentage of volunteers remaining qPCR negative (Kaplan–Meier survival proportions) after challenge infection with a homologous NF54 (n = 5 immunized; n = 5 controls) (**a**) or heterologous NF135.C10 (n = 10 immunized; n = 5 controls) (**b**) or NF166.C8 (n = 9 immunized; n = 5 controls) (**c**) strain is shown. Solid lines represent CPS-immunized volunteers and dotted lines represent placebo control-immunized volunteers. ** *P* < 0.01 as determined by Log-rank (Mantel Cox) test

As previously observed, NF54-CPS immunization induced sterile protection against a homologous NF54 challenge in 5 out of 5 volunteers (Fig. 2a) [11, 12, 32]. In contrast, 2 out of 10 volunteers were sterilely protected after challenge with NF135.C10, with 6 out of 10 volunteers showing a prolonged pre-patent period compared to mock-immunized controls (more than two-fold the standard deviation of controls) (Fig. 2b). After NF166.C8 challenge, 1 out of 9 NF54-immunized volunteers was fully protected, while 8 out of 9 volunteers showed no delay to patency (Fig. 2c). There was also no difference in mean day 7 parasitemia (representing the liver parasite burden) between immunized volunteers and controls challenged with NF166.C8 (Additional file 2: Figure S1). In line with a previous study [25], NF135.C10 and NF166.C8 showed higher infectivity than NF54. Overall, NF54-CPS immunization induced only modest protection against heterologous clones.

As a marker for the induction of immune responses, antibody levels to the major sporozoite vaccine-target antigen CSP [7, 8] were measured. All volunteers showed a post-immunization CSP antibody titer as determined by ELISAs and corrected for baseline (median 3.7 AU; interquartile range (IQR) 2.9–4.5), which did not significantly differ between groups (group 1: 24.7 (IQR 10.4–45.2), group 2: 24.7 (IQR 17.0–37.1), and group 3: 74.1 (IQR 20.9–187.0)).

### In vitro inhibition of intra-hepatic sporozoite development by CPS-induced antibodies

**Fig. 3:**
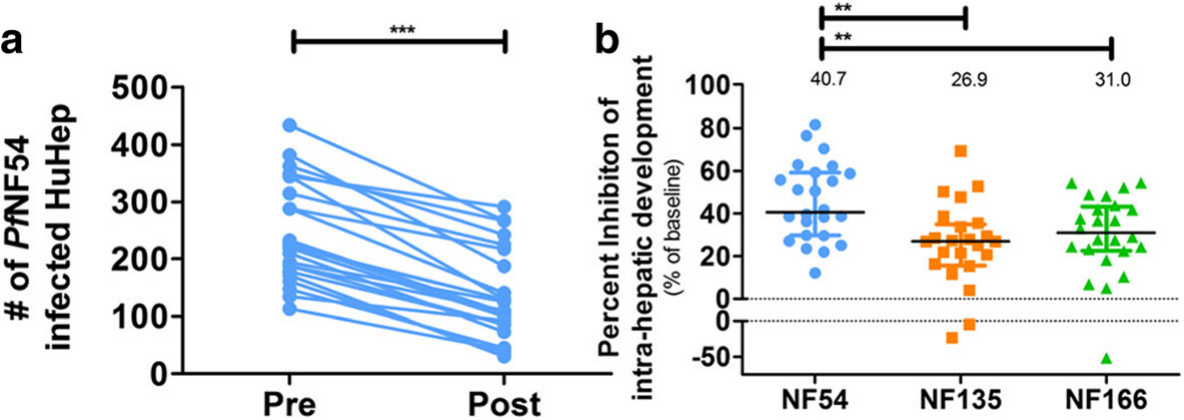
Neutralizing effect of CPS-induced antibodies on in vitro sporozoite functionality of homologous and heterologous *P. falciparum* strains. (**a**) The number of primary human hepatocytes infected by homologous NF54 sporozoites in the presence of pre- or post-immunization plasma in all (n = 24) CPS-immunized volunteers was determined by microscopy. (**b**) *P. falciparum* NF54, NF135.C10, or NF166.C8 sporozoites were pre-incubated with pre- or post-immunization plasma from CPS-immunized volunteers and the percent inhibition of intra-hepatic development of NF54, NF135.C10, or NF166.C8 was calculated for post- compared to pre-immunization plasma for each individual volunteer and presented as squares (NF135.C10), triangles (NF166.C8), or circles (NF54). Data are shown as the mean of triplicate measurements for each individual volunteer (**a**) or the median of all data points with an interquartile range (**b**). Differences in the percent inhibition of intra-hepatic development between parasite strains were tested using one-way ANOVA with Bonferroni’s multiple comparison correction

Next, functional antibody activity to inhibit sporozoite development in primary human hepatocytes in vitro was tested. Post-immunization plasma from all 24 NF54-CPS-immunized volunteers significantly reduced homologous intra-hepatic sporozoite development (*P* < 0.0001; Fig. 3a). However, inhibition of intra-hepatic NF135.C10 and NF166.C8 development was significantly lower (*P* < 0.01), with a median percentage inhibition of 40.7% (IQR 29.6–59.1), 26.9% (IQR 15.6–35.0), and 31.0% (IQR 22.6–43.2) for NF54, NF135.C10, and NF166.C8 sporozoites, respectively (Fig. 3b). Intra-hepatic development of both heterologous clones was equally inhibited by NF54-CPS immunization induced antibodies (Fig. 3b). While these in vitro data reflect the clinical outcome in vivo at group level, individual inhibition in vitro did not correlate with in vivo pre-patent periods and/or parasitemia (data not shown).

### Genetic diversity of NF54, NF135.C10, and N166.C8 challenge strains

**Fig. 4:**
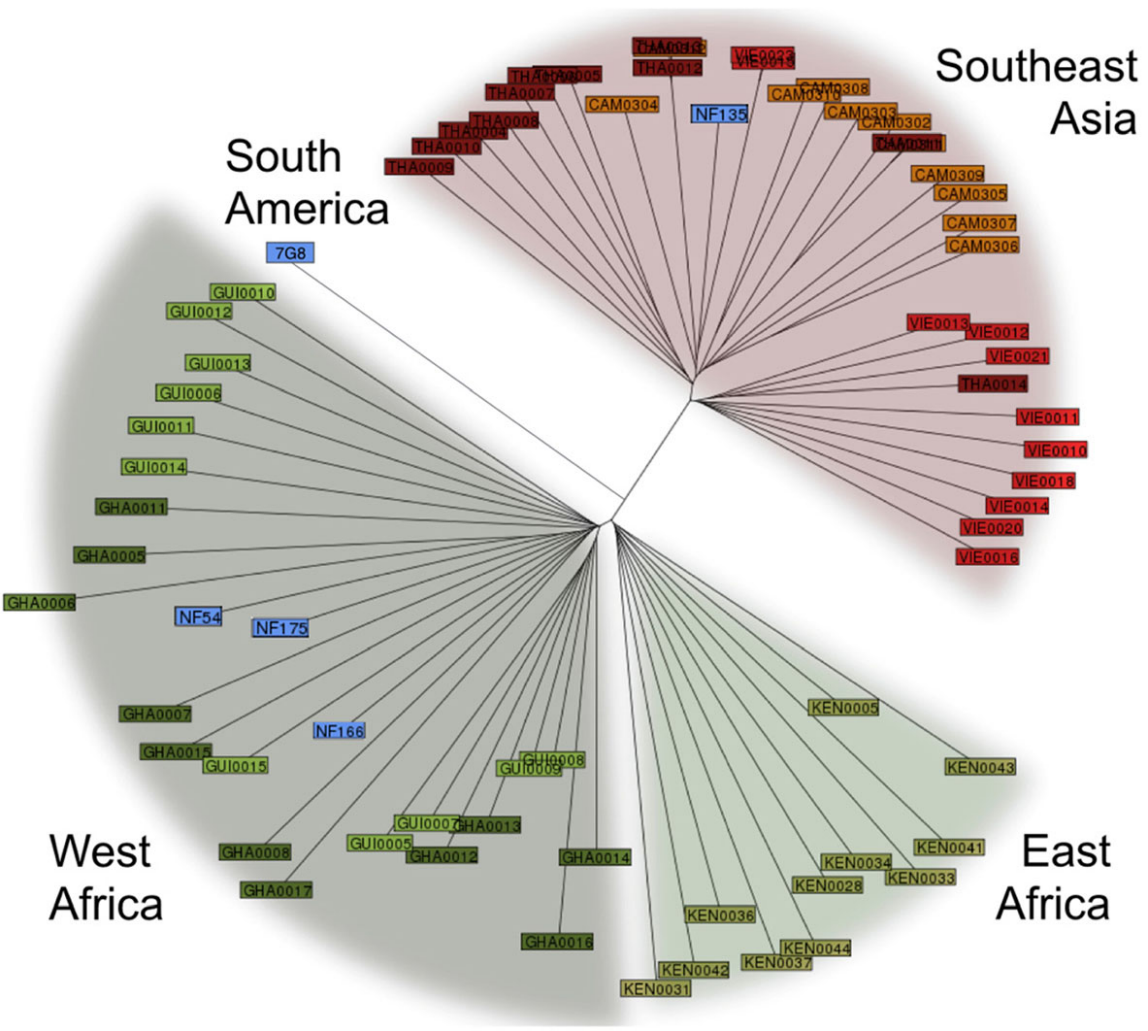
Whole-genome sequencing shows genetic variations between study strains. Phylogenetic positions of the three *P. falciparum* strains (NF54, NF135.C10 and NF166.C8) used in the study relative to other known *P. falciparum* strains. Whole-genome sequencing was used to infer relatedness to *P. falciparum* strains from different areas [31]. Asian strains: *THA* Thailand (dark red), *VIE* Vietnam (light red), *CAM* Cambodia (orange); East African strains: *KEN* Kenya; West African strains: *GUI* Guinea (light green), *GHA* Ghana (dark green); NF strains and 7G8 (blue)

The genetic diversity among *P. falciparum* mutants has been shown to be a strong factor in parasite evasion of protective immune responses. Whole genomes of the parasite clones used in this study were sequenced. High quality SNPs were called with about equal numbers for NF135.C10 and NF166.C8 (Additional file 2: Table S1; Figure S3). All polymorphisms were used to infer a phylogeny including isolates from Southeast Asia and East and West Africa [31]. As expected, NF54 and NF166.C8 were very similar to West African isolates, while NF135.C10 showed more resemblance to other Southeast Asian strains (Fig. 4). We next compared amino acid changes of 12 genes encoding target antigens for CPS-induced antibodies as previously described [16]. With the exception of EIF3A, all examined proteins showed amino acid changes in either NF135.C10 or NF166.C8 with respect to NF54 (Table 2). Remarkably, in contrast to their relative geographical distances, 11 out of 12 proteins in NF166.C8 and 8 out of 12 in NF135.C10 were different compared to NF54.

**Table 2:** Amino acid changes in genes involved in the humoral immune response after CPS immunization

Gene	*Pf*NF135.C10		*Pf*NF166.C8	
	Sequence changes	Protein changes	Sequence changes	Protein changes
PF3D7_1036400 LSA-1	6	4	14	9
PF3D7_0108300 Conserved unknown	21	12	15	8
PF3D7_1033100 AdoMetDC/ODC	10	9	9	8
PF3D7_0108300 MSP2	13	7	8	7
PF3D7_0304600 CSP	6	6 A98G S301N K317E E318Q N321K A361E	7	7 S301N K314Q K317E E318K N321K K322T E357Q
PF3D7_0509400 RNApol	6	4	9	6
PF3D7_0630600 Conserved unknown	7	2	8	3
PF3D7_0502400 MSP8	2	1	4	3
PF3D7_1147800 MAEBL	2	0	3	1
PF3D7_0703700 Conserved unknown	0	0	1	1
PF3D7_0829000 Conserved unknown	0	0	1	1
PF3D7_1212700 EIF3A	1	0	1	0

Alterations in gene and protein sequences, based on whole genome sequencing, for 12 genes from NF135.C10 and NF166.C8 were compared to NF54. Single nucleotide polymorphisms and small insertions/deletions are shown together as sequence changes with expected changes in the in silico-translated protein sequence (counting altered amino acids). Changes in CSP protein sequence are indicated with single-letter amino acid code

### Humoral and cellular markers of protection

**Fig. 5:**
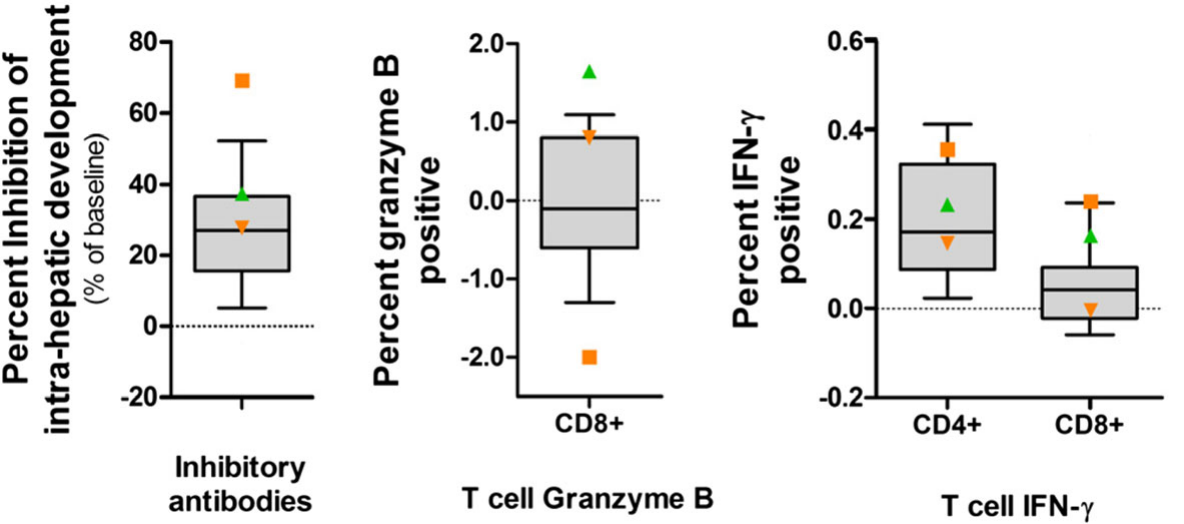
Analysis of in vitro intra-hepatic sporozoite development inhibition by CPS-induced antibodies, cellular responses and protection status in vivo. CPS-induced antibody-mediated inhibition of in vitro challenge strain intra-hepatic development and cytotoxic and cytokine-producing T cell responses to NF54-infected erythrocytes are shown. The 10th and 90th percentile of each response in all (*n* = 19) CPS-immunized volunteers that received a heterologous challenge infection are shown as grey box-and-whisker plots. The green triangle represents 1 out of 9 CPS-immunized volunteers sterilely protected against NF166.C8 challenge infection, while the orange square and upside down triangle represent the 2 out of 10 CPS-immunized volunteers with sterile protection against NF135.C10 challenge infection

Next, we tested a number of previously established markers associated with exposure and homologous protection after CPS immunization [11, 33]. Figure 5 shows the distribution of antibody-mediated inhibition of intra-hepatic sporozoite development as well as the cellular markers IFN-γ and granzyme B in CD4^+^ and CD8^+^ cells in all immunized volunteers undergoing heterologous challenge. Remarkably, CD107a expression, particularly in CD4^+^ T cells, was not increased as previously found (data not shown) [11]; 2 out of 3 heterologous protected volunteers were among the highest antibody-mediated inhibitors of in vitro heterologous intra-hepatic development (Fig. 5, orange square: NF135.C10-challenged; green triangle: NF166.C8-challenged), with the highest numbers of IFN-γ-producing CD8^+^ T cells against *Pf*RBC. The third protected volunteer showed only average neutralizing antibody responses and IFN-γ-producing CD4^+^ and CD8^+^ T cells, but had a high number of granzyme B^+^ CD8^+^ T cells (orange upside down triangle: NF135.C10-challenged). The group of NF135.C10 volunteers with a prolonged pre-patent period did not distinguish themselves from the unprotected volunteers.

## Discussion

This randomized, controlled clinical trial shows that CPS immunization with *P. falciparum* NF54 sporozoites induces modest sterile protection against challenge infection with genetically distinct *P. falciparum* parasites. In line with these clinical observations, antibodies induced by NF54-CPS immunization inhibit intra-hepatic development of both homologous and heterologous sporozoites in vitro, but less efficiently inhibit heterologous clones. *P. falciparum*-specific IFN-γ and granzyme B T cell responses are also induced, corroborating previous studies [11, 12], though unlike these studies, there was no clear induction of CD107a responses. We have previously shown that PBMCs from NF54-exposed volunteers have equivalent responses to homologous and heterologous *Pf*RBC stimulation [24]. Here, volunteers protected against heterologous challenge showed relatively high cellular and/or antibody responses. However, none of these individual markers predicts protection.

In this study, heterologous protection was assessed against a primary challenge infection in a double-blind manner, strongly reducing any potential for bias. However, this study lacks the power to discriminate the low protective efficacy seen in the NF166.C8 group with statistical significance. Furthermore, although we chose to test two geographically diverse heterologous strains, we do not know how representative these are for the total genetic diversity for *P. falciparum* in the field.

*P. falciparum* parasite strain diversity is a major impediment to the development of effective, sterilizing immunity [34]. Indeed, previous studies with single-protein vaccines show that antigen polymorphisms decrease vaccine efficacy [35–37]. However, genetic diversity may be less challenging for whole sporozoite vaccines representing a broader antigen repertoire that could increase the chances for generating functional, cross-strain immunity. In fact, immunization with radiation-attenuated sporozoites by 1000 mosquitoes [38] or with a total of 1.35 × 10^6^ sporozoites administered intravenously [39] has been shown to induce 80% protection against challenge with the heterologous challenge strain 7G8 (IMTM-22 isolate from Brazil) at 3 weeks post-immunization, rapidly waning to only 10% protection at 24 weeks [38, 39]. Doubling the dose of intravenous sporozoite immunization to a total of 2.7 × 10^6^ showed an adjusted 53% efficacy against 7G8 at 33 weeks [18]. These data indicate that induction of heterologous protection requires substantial high immunization dosages, which puts constraints on vaccine manufacturing and costs. In contrast, the CPS regimen shows a 10- to 20-fold higher efficiency for homologous protection, likely because volunteers are exposed to the full pre-erythrocytic cycle and the initial phase of the blood stage [40]. Previously, 2 out of 13 NF54-CPS-immunized volunteers receiving a sub-optimal immunization dose were protected against a re-challenge infection with NF135.C10 at 14 months after the last immunization [19]. Both studies with radiation-attenuated and CPS immunization showed higher homologous than heterologous protective efficacy. However, none of these studies used an immunization regimen sufficient for more than 90% homologous protective efficacy. The current study is the first randomized, controlled trial with a whole sporozoite immunization regimen sufficient for complete homologous protection, testing primary heterologous challenge infection 18 weeks after the last immunization [11].

Despite 100% protection against homologous challenge, we obtained only a modest 10–20% protection against heterologous strains. The observed difference in protection against homologous and heterologous infections may be explained by various factors. (1) Unequal distribution of induced immune responses between study groups; however, anti-CSP antibody titers show a clear consistency and similarity between study groups. (2) Differences in numbers of inoculation sporozoites or stringency of the challenge infection. The former is unlikely as mosquito salivary gland infections were similar between the generated strain batches (Additional file 2: Table S2). As NF135.C10 and NF166.C8 show higher sporozoite infectivity compared to NF54 [25], it may be more difficult to protect against the NF135.C10 and NF166.C8 clones. However, intra-hepatic sporozoite development of NF135.C10 and NF166.C8 was equipotently inhibited by a functional anti-CSP mAb, 2A10 (Additional file 2: Figure S2; IC50 concentrations of 2.5 μg/mL (95% CI 0.58–11 μg/mL), 3.5 μg/mL (95% CI 1.1–11 μg/mL), and 0.88 μg/mL (95% CI 0.26–2.9 μg/mL)), which makes this explanation less likely. (3) Genetic variation between the challenge parasites, shown to be considerable with amino acid changes in either NF135.C10 or NF166.C8 in 11 out of 12 target proteins for CPS-induced antibodies [16]. We consider decreased immune efficacy against genetically diverse parasite strains as the most likely explanation for the modest protection against heterologous parasites.

Since 3 out of 19 volunteers with sterile protection against heterologous challenge tended to show more potent responses to previously identified immune markers [11, 15, 33], decreased efficacy against heterologous parasites may be overcome by stronger immune responses. Improvement of the strength of cellular and antibody responses may be achieved by altering immunization regimens. For instance, this may be achieved by increasing the immunization liver-stage antigen load by raising the NF54 sporozoite immunization dose, as has been done with radiation-attenuated sporozoites [18, 41]. Alternatively, increased liver-stage infectivity with a parasite such as NF135.C10 likely increases the antigen load without the need to increase the number of sporozoites administered. A mixture of parasite strains or sequential immunizations with different strains are alternative options, but will complicate the product manufacturing or its practical application. The latter approach might be more efficient, as heterologous sporozoites would evade strain-specific neutralizing immunity, thereby increasing the liver parasite burden at second and third immunizations. These studies should aim to find the optimal balance between the desired induction of cross-stage immunity and the related adverse events that may occur. Taken together, our findings highlight the need to further explore the immunological basis of cross-strain protection against *P. falciparum* in order to improve existing whole-sporozoite immunization strategies.

## Conclusions

These data demonstrate that, despite providing complete protection against homologous challenge infection, CPS immunization with the NF54 strain provides only modest sterile protection against the genetically distinct NF135.C10 and NF166.C8 clones. Our immunological and parasite sequencing data suggest that genetic variation between the strains reduces the efficiency of antibodies to block heterologous parasites. Since protected volunteers tended to be higher immune responders, this study underscores the need for whole sporozoite vaccination regimens to increase the height or breadth of immune responses to achieve heterologous protection.

## Additional files

Additional file 1: Protocol. Malaria antigen-specific IgG ELISA (S1). Isolation and cultivation of primary human hepatocytes (S2). In vitro sporozoite infectivity assay of primary human hepatocytes (S3). Immunofluorescent analysis of P. falciparum-infected primary human hepatocytes (S4). In vitro PBMC restimulation with PfRBCs and flow cytometry staining (S5) [11, 12, 14, 17, 42]. (DOCX 20 kb)

Additional file 2: Figure S1. First-wave parasitemia after challenge. Parasitemia on day 7 post-challenge in immunized (open circles) and control (closed circles) volunteers. The line and error bars show the geometric mean and 95% CI interval. **Figure S2.** Inhibition of in vitro homologous and heterologous intra-hepatic sporozoite development in primary human hepatocytes by mAb 2A10. *P. falciparum* NF54 (blue), NF135.C10 (orange) and NF166.C8 (green) sporozoites in the presence of 10% heat-inactivated naive human control serum were pre-incubated with 3-fold serial dilutions of the 2A10 monoclonal antibody (0.027–20 μg/mL), targeting the repeat region of the circumsporozoite protein (CSP), and added to primary human hepatocyte cultures. Six days post-infection, the number of *P. falciparum*-infected hepatocytes was assessed as described in Additional file 1: S4 and S5. **Figure S3.** Amino acid changes in CSP. **Table S1.** Whole-genome sequencing statistics. **Table S2.** Mosquito salivary gland infectivity and sporozoite load of the three clones. Mean mosquito salivary gland infectivity and sporozoite load determined 1 day prior to challenge infection by dissecting a sample of 10 mosquitoes per strain. (DOCX 427 kb)

## Abbreviations

95% CI: 95% confidence interval
BMI: body mass index
CHMI: controlled human malaria infection
CPS: chemoprophylaxis and sporozoite immunization
CSP: circumsporozoite protein
CVD: cardiovascular disease
ECG: electrocardiogram
ELISA: enzyme-linked immunosorbent assay
IFN-γ: interferon gamma
IQR: interquartile range
mAb: monoclonal antibody
*P. falciparum*: *Plasmodium falciparum*
PBMCs: peripheral blood mononuclear cells
qPCR: quantitative real-time PCR
SNPs: single nucleotide polymorphisms

## References

[1] World Health Organization (2015). World Malaria Report 2015.

[2] Hemingway J, Ranson H (2000). Insecticide resistance in insect vectors of human disease. Annu Rev Entomol.

[3] Ashley EA, Dhorda M, Fairhurst RM, Amaratunga C, Lim P, Suon S, Sreng S, Anderson JM, Mao S, Sam B (2014). Spread of artemisinin resistance in Plasmodium falciparum malaria. N Engl J Med.

[4] Tran TM, Li S, Doumbo S, Doumtabe D, Huang CY, Dia S, Bathily A, Sangala J, Kone Y, Traore A (2013). An intensive longitudinal cohort study of Malian children and adults reveals no evidence of acquired immunity to Plasmodium falciparum infection. Clin Infect Dis.

[5] European Medicines Agency. First malaria vaccine receives positive scientific opinion from EMA. Mosquirix to be used for vaccination of young children, together with established antimalarial interventions. 2015. http://www.ema.europa.eu/ema/index.jsp?curl=pages/news_and_events/news/2015/07/news_detail_002376.jsp&mid=WC0b01ac058004d5c1. Accessed 17 Aug 2017.

[6] Kester KE, Cummings JF, Ofori-Anyinam O, Ockenhouse CF, Krzych U, Moris P, Schwenk R, Nielsen RA, Debebe Z, Pinelis E (2009). Randomized, double-blind, phase 2a trial of falciparum malaria vaccines RTS, S/AS01B and RTS, S/AS02A in malaria-naive adults: safety, efficacy, and immunologic associates of protection. J Infect Dis.

[7] Moorthy VS, Okwo-Bele JM (2015). Final results from a pivotal phase 3 malaria vaccine trial. Lancet.

[8] Olotu A, Fegan G, Wambua J, Nyangweso G, Awuondo KO, Leach A, Lievens M, Leboulleux D, Njuguna P, Peshu N (2013). Four-year efficacy of RTS, S/AS01E and its interaction with malaria exposure. N Engl J Med.

[9] Hickey BW, Lumsden JM, Reyes S, Sedegah M, Hollingdale MR, Freilich DA, Luke TC, Charoenvit Y, Goh LM, Berzins MP (2016). Mosquito bite immunization with radiation-attenuated Plasmodium falciparum sporozoites: safety, tolerability, protective efficacy and humoral immunogenicity. Malar J.

[10] Seder RA, Chang LJ, Enama ME, Zephir KL, Sarwar UN, Gordon IJ, Holman LA, James ER, Billingsley PF, Gunasekera A (2013). Protection against malaria by intravenous immunization with a nonreplicating sporozoite vaccine. Science.

[11] Bijker EM, Teirlinck AC, Schats R, van Gemert GJ, van de Vegte-Bolmer M, van Lieshout L, IntHout J, Hermsen CC, Scholzen A, Visser LG (2014). Cytotoxic markers associate with protection against malaria in human volunteers immunized with Plasmodium falciparum sporozoites. J Infect Dis.

[12] Roestenberg M, McCall M, Hopman J, Wiersma J, Luty AJ, van Gemert GJ, van de Vegte-Bolmer M, van Schaijk B, Teelen K, Arens T (2009). Protection against a malaria challenge by sporozoite inoculation. N Engl J Med.

[13] Mordmuller B, Surat G, Lagler H, Chakravarty S, Ishizuka AS, Lalremruata A, Gmeiner M, Campo JJ, Esen M, Ruben AJ (2017). Sterile protection against human malaria by chemoattenuated PfSPZ vaccine. Nature.

[14] Roestenberg M, Teirlinck AC, McCall MB, Teelen K, Makamdop KN, Wiersma J, Arens T, Beckers P, van Gemert G, van de Vegte-Bolmer M (2011). Long-term protection against malaria after experimental sporozoite inoculation: an open-label follow-up study. Lancet.

[15] Behet MC, Foquet L, van Gemert GJ, Bijker EM, Meuleman P, Leroux-Roels G, Hermsen CC, Scholzen A, Sauerwein RW (2014). Sporozoite immunization of human volunteers under chemoprophylaxis induces functional antibodies against pre-erythrocytic stages of Plasmodium falciparum. Malar J.

[16] Felgner PL, Roestenberg M, Liang L, Hung C, Jain A, Pablo J, Nakajima-Sasaki R, Molina D, Teelen K, Hermsen CC (2013). Pre-erythrocytic antibody profiles induced by controlled human malaria infections in healthy volunteers under chloroquine prophylaxis. Sci Rep.

[17] Nahrendorf W, Scholzen A, Bijker EM, Teirlinck AC, Bastiaens GJ, Schats R, Hermsen CC, Visser LG, Langhorne J, Sauerwein RW (2014). Memory B-cell and antibody responses induced by Plasmodium falciparum sporozoite immunization. J Infect Dis.

[18] Lyke KE, Ishizuka AS, Berry AA, Chakravarty S, DeZure A, Enama ME, James ER, Billingsley PF, Gunasekera A, Manoj A (2017). Attenuated PfSPZ Vaccine induces strain-transcending T cells and durable protection against heterologous controlled human malaria infection. Proc Natl Acad Sci U S A.

[19] Schats R, Bijker EM, van Gemert GJ, Graumans W, van de Vegte-Bolmer M, van Lieshout L, Haks MC, Hermsen CC, Scholzen A, Visser LG (2015). Heterologous protection against malaria after immunization with Plasmodium falciparum sporozoites. PLoS One.

[20] Bijker EM, Schats R, Obiero JM, Behet MC, van Gemert GJ, van de Vegte-Bolmer M, Graumans W, van Lieshout L, Bastiaens GJ, Teelen K (2014). Sporozoite immunization of human volunteers under mefloquine prophylaxis is safe, immunogenic and protective: a double-blind randomized controlled clinical trial. PLoS One.

[21] Hermsen CC, Telgt DS, Linders EH, van de Locht LA, Eling WM, Mensink EJ, Sauerwein RW (2001). Detection of Plasmodium falciparum malaria parasites in vivo by real-time quantitative PCR. Mol Biochem Parasitol.

[22] Walk J, Schats R, Langenberg MC, Reuling IJ, Teelen K, Roestenberg M, Hermsen CC, Visser LG, Sauerwein RW (2016). Diagnosis and treatment based on quantitative PCR after controlled human malaria infection. Malar J.

[23] Delemarre BJ, van der Kaay HJ (1979). Tropical malaria contracted the natural way in the Netherlands. Ned Tijdschr Geneeskd.

[24] Teirlinck AC, Roestenberg M, van de Vegte-Bolmer M, Scholzen A, Heinrichs MJ, Siebelink-Stoter R, Graumans W, van Gemert GJ, Teelen K, Vos MW (2013). NF135.C10: a new Plasmodium falciparum clone for controlled human malaria infections. J Infect Dis.

[25] McCall MBB, Wammes LJ, Langenberg MCC, van Gemert GJ, Walk J, Hermsen CC, Graumans W, Koelewijn R, Franetich JF, Chishimba S, et al. Infectivity of Plasmodium falciparum sporozoites determines emerging parasitemia in infected volunteers. Sci Transl Med. 2017;9(395). 10.1126/scitranslmed.aag2490 28637923

[26] Ifediba T, Vanderberg JP (1981). Complete in vitro maturation of Plasmodium falciparum gametocytes. Nature.

[27] Ponnudurai T, Lensen AH, Leeuwenberg AD, Meuwissen JH (1982). Cultivation of fertile Plasmodium falciparum gametocytes in semi-automated systems. 1. Static cultures. Trans R Soc Trop Med Hyg.

[28] Ponnudurai T, Lensen AH, Meis JF, Meuwissen JH (1986). Synchronization of Plasmodium falciparum gametocytes using an automated suspension culture system. Parasitology.

[29] Ponnudurai T, Lensen AH, Van Gemert GJ, Bensink MP, Bolmer M, Meuwissen JH (1989). Infectivity of cultured Plasmodium falciparum gametocytes to mosquitoes. Parasitology.

[30] Kastenmuller et al. Full-Length Plasmodium falciparum Circumsporozoite Protein Administered with Long-Chain Poly(I·C) or the Toll-Like Receptor 4 Agonist Glucopyranosyl Lipid Adjuvant-Stable Emulsion Elicits Potent Antibody and CD4 T Cell Immunity and Protection in Mice. Infection and Immunity. 2013;81(3):789–800. 10.1128/IAI.01108-12 PMC3584875 23275094

[31] Campino S, Benavente ED, Assefa S, Thompson E, Drought LG, Taylor CJ, Gorvett Z, Carret CK, Flueck C, Ivens AC (2016). Genomic variation in two gametocyte non-producing Plasmodium falciparum clonal lines. Malar J.

[32] Bijker EM, Bastiaens GJ, Teirlinck AC, van Gemert GJ, Graumans W, van de Vegte-Bolmer M, Siebelink-Stoter R, Arens T, Teelen K, Nahrendorf W (2013). Protection against malaria after immunization by chloroquine prophylaxis and sporozoites is mediated by preerythrocytic immunity. Proc Natl Acad Sci U S A.

[33] McCall MB, Sauerwein RW (2010). Interferon-gamma--central mediator of protective immune responses against the pre-erythrocytic and blood stage of malaria. J Leukoc Biol.

[34] Doolan DL, Dobano C, Baird JK (2009). Acquired immunity to malaria. Clin Microbiol Rev.

[35] Neafsey DE, Juraska M, Bedford T, Benkeser D, Valim C, Griggs A, Lievens M, Abdulla S, Adjei S, Agbenyega T (2015). Genetic diversity and protective efficacy of the RTS, S/AS01 malaria vaccine. N Engl J Med.

[36] Takala SL, Coulibaly D, Thera MA, Dicko A, Smith DL, Guindo AB, Kone AK, Traore K, Ouattara A, Djimde AA (2007). Dynamics of polymorphism in a malaria vaccine antigen at a vaccine-testing site in Mali. PLoS Med.

[37] Thera MA, Doumbo OK, Coulibaly D, Laurens MB, Ouattara A, Kone AK, Guindo AB, Traore K, Traore I, Kouriba B (2011). A field trial to assess a blood-stage malaria vaccine. N Engl J Med.

[38] Hoffman SL, Goh LM, Luke TC, Schneider I, Le TP, Doolan DL, Sacci J, de la Vega P, Dowler M, Paul C (2002). Protection of humans against malaria by immunization with radiation-attenuated Plasmodium falciparum sporozoites. J Infect Dis.

[39] Epstein JE, Paolino KM, Richie TL, Sedegah M, Singer A, Ruben AJ, Chakravarty S, Stafford A, Ruck RC, Eappen AG (2017). Protection against Plasmodium falciparum malaria by PfSPZ vaccine. JCI Insight.

[40] Nganou-Makamdop K, Sauerwein RW (2013). Liver or blood-stage arrest during malaria sporozoite immunization: the later the better?. Trends Parasitol.

[41] Epstein JE, Tewari K, Lyke KE, Sim BK, Billingsley PF, Laurens MB, Gunasekera A, Chakravarty S, James ER, Sedegah M (2011). Live attenuated malaria vaccine designed to protect through hepatic CD8(+) T cell immunity. Science.

[42] Verhave JP, Leeuwenberg AD, Ponnudurai T, Meuwissen JH, van Druten JA (1988). The biotin-streptavidin system in a two-site ELISA for the detection of plasmodial sporozoite antigen in mosquitoes. Parasite Immunol.

