# Supplementary material for: Modest heterologous protection after Plasmodium falciparum sporozoite immunization: a double-blind randomized controlled clinical trial
Source: BMC Med. 2017 Sep 13;15:168. doi: 10.1186/s12916-017-0923-4 (PMC5598044; doi:10.1186/s12916-017-0923-4)
Supplement: Supplementary file 1 — Protocol. Malaria antigen-specific IgG ELISA (S1). Isolation and cultivation of primary human hepatocytes (S2). In vitro sporozoite infectivity assay of primary human hepatocytes (S3). Immunofluorescent analysis of P. falciparum-infected primary human hepatocytes (S4). In vitro PBMC restimulation with PfRBCs and flow cytometry staining (S5) [11, 12, 14, 17, 42]. (DOCX 20 kb) [file 12916_2017_923_MOESM1_ESM.docx]

**Additional file 1**

**Protocol: malaria antigen-specific IgG ELISA (S1)**

Levels of malaria antigen-specific antibodies in plasma were determined by a standardized enzyme-linked immunosorbent assay (ELISA) and expressed as arbitrary units (AU) in relation to a pool of 100 sera from adults living in an area in Tanzania where malaria is highly endemic [12].

Polystyrene flat-bottom 96 well plates (ThermoScientific, NUNC^TM^ Maxisorp, 439454) were coated overnight at 4 °C with 0.5 µg/ml circumsporozoite protein (CSP, Genova) in PBS. Subsequently, plates were washed four times with PBS, blocked with 5% milk in PBS for 1 h at room temperature and again washed four times with PBS. Citrated plasma samples were diluted 1:50 in PBS containing 0.05% Tween20 and 1% milk (PBST/1% milk), analyzed in duplicate and a three-point 1:3 dilution series was carried out for each plasma sample. Both time points from each volunteer were measured on the same plate. As a standard and positive control, a plasma pool obtained from 100 Tanzanian hyper-immune adults living in a highly malaria-endemic area (HIT) was diluted 1:200 in PBST/1% milk and included on every plate in a seven-point 1:2 dilution series in duplicate. After 3h incubation with plasma samples at room temperature, plates were washed four times with PBST and then four times with PBS. Bound malaria antigen-specific IgG antibodies were detected by incubation with a 1:40,000 dilution of horseradish peroxidase (HRP)-conjugated polyclonal rabbit anti-human IgG antibody (P0214, Dako Denmark) for 1h at room temperature, followed by washing four times with PBST and PBS. Plates were developed with HRP substrate (tetra-methyl-benzidine, tetu-bio laboratories) at room temperature. The reaction was stopped using 0.2M H_2_SO_4_ after 13 minutes. Spectrophotometrical absorbance at 450 nm was measured using the iMark Microplate Absorbance Reader (Bio-Rad). ELISA data analysis was performed with Auditable Data Analysis and Management System for ELISA (ADAMSEL, version 1.1) as previously described [17]. Antibody levels were calculated in relation to the positive control (HIT plasma pool), which was set at 100 arbitrary units (AU).

**Protocol: Isolation and cultivation of primary human hepatocytes (S2)**

Fresh primary human hepatocytes were isolated from patients undergoing partial hepatectomy as described. Remnant liver tissue, varying 1-5cm^3^ in size, was transferred into a 1000 ml sterile glass containing 100 ml UW (Bristol-Meyers-Squibb, 1148162) immediately after isolation from the patient and transported to the laboratory. The liver tissue was put on sterile bandage gauze and perfused via any vessel (venous or portal vein) with 500 ml of oxygenized HBSS medium (Gibco, 14170-088) supplemented with 10mM HEPES (Gibco, 15630-056) and 0.64 mM EDTA (Invitrogen, 15575-038) to inactivate proteases. Subsequently, the liver tissue was perfused with 500 ml of oxygenized HBSS medium supplemented with 10mM HEPES, followed by perfusion with 100 ml of oxygenized HBSS medium supplemented with 10mM HEPES, 0.75 mg/ml CaCl_2_ and low concentrations of collagenase (3,333 units per 50ml). Next, the liver tissue was perfused with 100 ml of oxygenized HBSS medium supplemented with 10mM HEPES, 0.75 mg/ml CaCl_2_ and high concentrations of collagenase (13,333 units per 50ml). This buffer was re-used for perfusion for maximum 20 minutes until the liver tissue became very soft. Subsequently, the liver tissue was transferred into a petri dish containing 40 ml cold DMEM medium (Gibco, 31885-023) supplemented with 10% FBS (Gibco, 10270) to inactivate collagenase activity. The liver tissue was cut into small pieces and clouds of hepatocytes flowing into the medium could be observed. Medium containing hepatocytes was transferred into a 50ml Falcon tube over a 100 µm cell strainer (Falcon, 352360) and meanwhile, another 40 ml of cold DMEM supplemented with 10% FBS was added to the small liver pieces. Medium was again collected and run over a 100 µm cell strainer. Subsequently, primary human hepatocytes were centrifuged at 10g with low brake for 5 minutes at 4 °C. Hepatocyte pellets were washed in cold DMEM without serum and again centrifuged at 10g with low brake for 5 minutes at 4 °C. This step was repeated until the supernatant looked clear. Subsequently, cells were resuspended in 30 ml complete William's B medium, consisting of William's E medium with Glutamax (Gibco, 32551-087) supplemented with 10% heat-inactivated human serum, 1% insulin/transferrin/selenium (Gibco, 41400-045), 1% sodium pyruvate (Gibco, 11360-036), 1% MEM-NEAA (Gibco, 1140-035), 1% Fungizone Antimycotic (Gibco, 15290-018), 2% penicillin/streptomycin (Gibco, 15140-122) and 1.6 µM dexamethasone, and 15 ml each was carefully stacked upon 25ml of 25% Percoll in 50 ml Falcon tubes. Tubes were centrifuged at 2,000 RPM without brake for 2 minutes at 4 °C. Supernatant containing dead cells was carefully removed and the pellet was resuspended in 5-10 ml complete William's B medium and counted at a 1:2 dilution in Trypan blue. Viable primary human hepatocytes were diluted to a final concentration of 0.5x10^6^ hepatocytes/ml in complete William's B medium and 5.10^4^ primary human hepatocytes were seeded into each well in black 96 well plates (Falcon, 353219) coated with 0.056 mg/ml rat tail collagen I/well (Roche Applied Science, 11179179-001). Hepatocytes were cultured at 37 °C in an atmosphere of 5% CO_2_, medium was refreshed the next morning (100 µl/well complete William's B medium) and then every two days.

**Protocol: *In vitro* sporozoite infectivity assay of primary human hepatocytes (S3)**

Two to three days following seeding of primary human hepatocytes into 96 well plates, the neutralizing activity of CPS-induced antibodies on *in vitro* sporozoite invasion was assessed in *in vitro* sporozoite infectivity assays with primary human hepatocytes.

*P. falciparum* NF54, NF135.C10 or NF166.C8 sporozoites were pre-incubated with 10% heat-inactivated naive control human serum and 10% heat-inactivated plasma from CPS-immunized volunteers (either Pre- or Post-immunization plasma) for 30 minutes on ice. Sporozoites pre-incubated with 20% heat-inactivated non-immune human serum in the presence or absence of 10 µg/ml of monoclonal anti-CSP antibody [42] served as a positive and standard control, respectively.

5·10^4^ sporozoites were added to 96 well plates containing monolayers of 5·10^4^ primary human hepatocytes in triplicates, centrifuged at 3,000 RPM for 10 minutes at RT with a low brake (Eppendorf Centrifuge 5810 R) and incubated for 3 hours at 37 °C in 5% CO_2_. After incubation, medium containing sporozoites was gently removed to remove non-invaded sporozoites and subsequently, 100 µl/well complete William's B medium was added to each well. Plates were kept at 37°C in 5% CO_2_ and medium of primary human hepatocytes was refreshed every two days. Five to six days after infection of primary human hepatocytes with *P. falciparum* sporozoites, wells were gently washed three times with PBS and fixed with 4% paraformaldehyde for 15-20 minutes at room temperature. After fixation, wells were gently washed three times with PBS and 200 µl PBS was added to each well. Plates were stored at 4 °C in the dark until immuno-fluorescent staining of infected primary human hepatocytes. Investigators carrying out the *in vitro* sporozoite infectivity assay of primary human hepatocytes were blinded to the volunteer identification codes and their respective outcomes during immunization and following challenge infection.

**Protocol: Immunofluorescent analysis of *P. falciparum*-infected primary human hepatocytes (S4)**

The number of primary human hepatocytes harbouring *P. falciparum* parasites was assessed by indirect immuno-fluorescence analysis. Firstly, aldehyde groups in hepatocyte cultures were blocked with 100 µl/well 0·1M glycine for 10 minutes at room temperature to prevent autofluorescence and hepatocytes were gently washed three times with 200 µl/well PBS. Subsequently, non-specific protein binding sites in the wells were blocked with 100 µl/well 10% FBS in PBS for 20 minutes at room temperature and wells were gently washed three times with 200 µl/well PBS. Non-invaded or adhering sporozoites were stained with 50 µl/well of an anti-CSP antibody conjugated with FITC (1:50 diluted in 10% FBS in PBS) for 2 hours at room temperature in the dark. Hepatocytes were gently washed three times with 200 µl/well PBS and subsequently permeabilized for 5 minutes at room temperature with 1% Triton X-100 (Sigma T9284, diluted in PBS). Primary human hepatocytes infected with viable parasites were stained intracellularly with 50 µl/well of anti-*P. falciparum* Hsp-70 rabbit polyclonal antibody (StressMarQ BioSciences, SPC-186D; 1:75 dilution in 10% FBS in PBS) for 2 hours at room temperature in the dark. Hepatocytes were gently washed three times with 200 µl/well PBS and subsequently stained with a secondary antibody and a nuclei stain (Alexa Fluor 594 goat-anti-rabbit antibody and DAPI nuclei stain, 1:200 and 1:100 diluted in 10% FBS in PBS, respectively). Hepatocytes were gently washed three times with 200 µl/well PBS and finally, 200 µl PBS/well was added to each well. Plates were stored at 4 °C in the dark until fluorescent microscopic analysis.

Overview images of each well were captured using a Leica DMI6000B inverted microscope and adjusted for brightness and/or contrast using the ImageJ 1.48v program (NIH, USA). Channels (red, green and blue) were split and a macro that was made for automatically counting of NF54, NF135 or NF166 parasites in primary human hepatocytes, was run on the picture from the red channel. To check the sensitivity of the macro, selected wells were also manually counted for the presence of parasites using the ImageJ 1.48v program (NIH, USA). Differences between automatical and manual counts of no more than 10% were accepted.

**Protocol: *In vitro* PBMC restimulation with *Pf*RBCs and flow cytometry staining (S5)**

Pre- and post-immunization PBMCs from CPS-immunized volunteers whom received NF135.C10 and NF166.C8-challenge infection were restimulated *in vitro* with cryopreserved *P. falciparum* NF54-infected erythrocytes (*Pf*RBCs) as described previously [43, 11]. PBMCs were thawed and washed twice with Dutch Modified RPMI 1640 (Gibco) and counted in 0·1% Trypan blue with 5% Zap-o-Globin II Lytic Reagent (Beckman Coulter) to assess cell viability. Cells were resuspended in complete culture medium (Dutch Modified RPMI 1640 containing 2 mM glutamine, 1mM pyruvate and 0·05 mM gentamycin and 10% human A+ serum (Sanquin, Nijmegen) at a final concentration of 10*10^6^cells/ml. 0·5*10^6^ PBMCs/well were transferred into 96-well round-bottom plates and stimulated *in vitro* in duplicate with either 10^6^ cryopreserved NF54 *Pf*RBCs or 10^6^ uninfected erythrocytes (uRBC) for 24h at 37 °C with 5% CO_2_. During the last four hours, 10 µg/ml Brefeldin A (Sigma-Aldrich) and 2 µM monensin (eBioscience) were added, and 10 ng/ml PMA (Sigma-Aldrich) and 1 µg/ml ionomycin (Sigma-Aldrich) were added to positive control wells. After 24 hours of stimulation in total, cells were harvested and stained. PBMCs were first stained with Live/Dead fixable dead cell stain dye eF780 (eBioscience) and after washing, cells were stained with antibodies against the surface markers CD3 ECD (Beckman Coulter; clone UCHT1), CD4 V500 (BD Horizon; clone RPA-T4), CD8 AF700 (BioLegend; clone HIT8A), gamma delta T cell receptor PE (Beckman Coulter; clone IMMU510) and CD56 PerCP/Cy5·5 (BioLegend; clone HCD56). Cells were washed again, fixed in Foxp3 fixation/permeabilization buffer (eBioscience) and stained with antibodies against the intracellular cytotoxic marker granzyme B FITC (BioLegend; clone GB11) and the cytokine IFNƴ PECy7 (BioLegend; clone 4S.B3). After washing cells in permeabilization buffer, samples were kept cold and dark in 1% paraformaldehyde (PFA) in PBS until measured by flow cytometry on the same day. Both time points each volunteer were thawed, stimulated and stained within the same experimental round. Samples were acquired using a 10-colour Gallios flow cytometer (Beckman Coulter), and single stained cells were run every round for compensation. Data analysis was performed using FlowJo software (Version 10·0·8, Tree Star). uRBC responses were substracted from *Pf*RBC-specific responses for every volunteer on every time point, and post-immunization responses were corrected for pre-immunization responses.

11. Bijker, E.M., et al., *Cytotoxic markers associate with protection against malaria in human volunteers immunized with Plasmodium falciparum sporozoites.* J Infect Dis, 2014. **210**(10): p. 1605-15.

12. Roestenberg, M., et al., *Protection against a malaria challenge by sporozoite inoculation.* N Engl J Med, 2009. **361**(5): p. 468-77.

17. Nahrendorf, W., et al., *Memory B-cell and antibody responses induced by Plasmodium falciparum sporozoite immunization.* J Infect Dis, 2014. **210**(12): p. 1981-90.

42. Verhave, J.P., et al., *The biotin-streptavidin system in a two-site ELISA for the detection of plasmodial sporozoite antigen in mosquitoes.* Parasite Immunol, 1988. **10**(1): p. 17-31.

43. Teirlinck, A.C., et al., *Longevity and composition of cellular immune responses following experimental Plasmodium falciparum malaria infection in humans.* PLoS Pathog, 2011. **7**(12): p. e1002389.
