# Supplementary material for: Modest heterologous protection after Plasmodium falciparum sporozoite immunization: a double-blind randomized controlled clinical trial
Source: BMC Med. 2017 Sep 13;15:168. doi: 10.1186/s12916-017-0923-4 (PMC5598044; doi:10.1186/s12916-017-0923-4)
Supplement: Supplementary file 2 — First-wave parasitemia after challenge. Parasitemia on day 7 post-challenge in immunized (open circles) and control (closed circles) volunteers. The line and error bars show the geometric mean and 95% CI interval. Figure S2. Inhibition of in vitro homologous and heterologous intra-hepatic sporozoite development in primary human hepatocytes by mAb 2A10. P. falciparum NF54 (blue), NF135.C10 (orange) and NF166.C8 (green) sporozoites in the presence of 10% heat-inactivated naive human control serum were pre-incubated with 3-fold serial dilutions of the 2A10 monoclonal antibody (0.027–20 μg/mL), targeting the repeat region of the circumsporozoite protein (CSP), and added to primary human hepatocyte cultures. Six days post-infection, the number of P. falciparum-infected hepatocytes was assessed as described in Additional file 1: S4 and S5. Figure S3. Amino acid changes in CSP. Table S1. Whole-genome sequencing statistics. Table S2. Mosquito salivary gland infectivity and sporozoite load of the three clones. Mean mosquito salivary gland infectivity and sporozoite load determined 1 day prior to challenge infection by dissecting a sample of 10 mosquitoes per strain. (DOCX 427 kb) [file 12916_2017_923_MOESM2_ESM.docx]

**Additional file 2**

**Figure S1: First-wave parasitemia after challenge.**

Parasitemia on day 7 post challenge in immunized (open circles) and control (closed circles) volunteers. The line and error bars show the geometric mean and 95% CI interval.

**
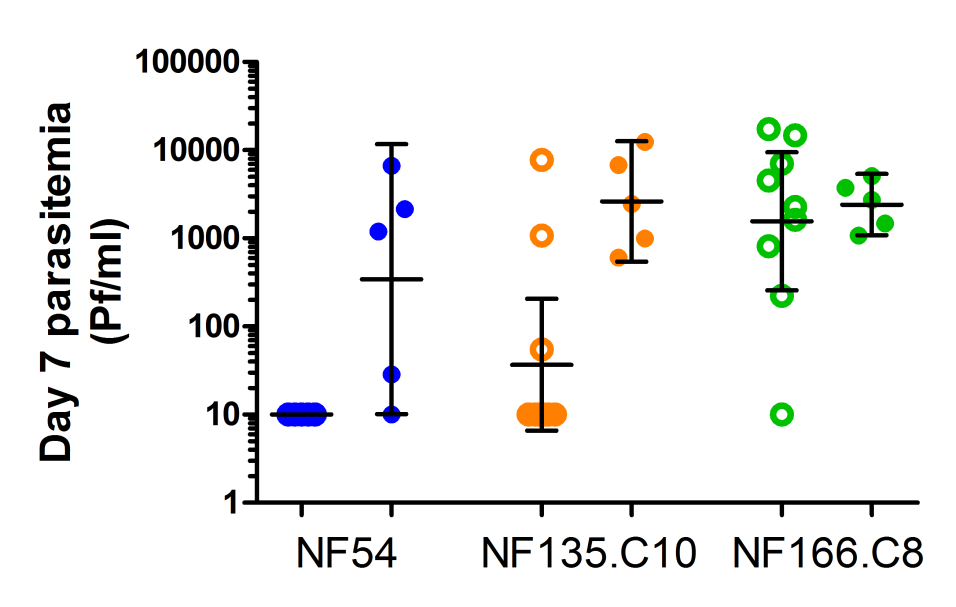
**

**Figure S2: Inhibition of *in vitro* homologous and heterologous intra-hepatic sporozoite development in primary human hepatocytes by mAb 2A10.**

*P. falciparum* NF54 (blue), NF135.C10 (orange) and NF166.C8 (green) sporozoites in the presence of 10% heat-inactivated naive human control serum were pre-incubated with 3-fold serial dilutions of the 2A10 monoclonal antibody (0.027-20 µg/ml), targeting the repeat region of the circumsporozoite protein (CSP), and added to primary human hepatocyte cultures. Six days post-infection, the number of *P. falciparum* infected hepatocytes was assessed as described in Supplementary information S4 and S5.

**
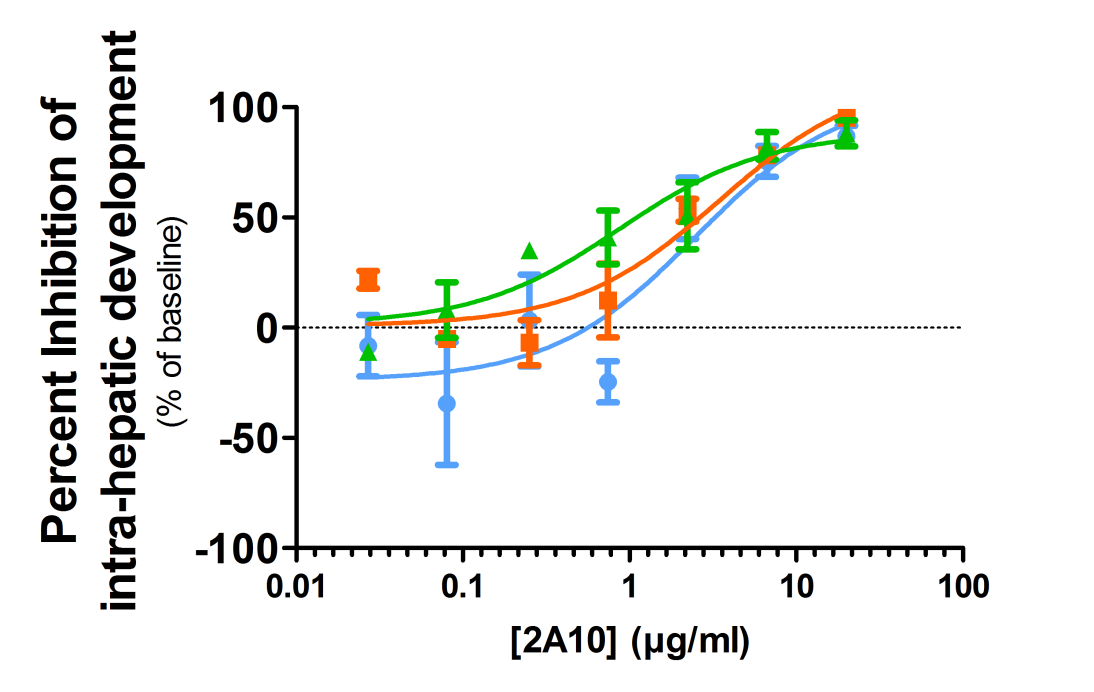
**

**Figure S3: Amino acid changes in CSP.**


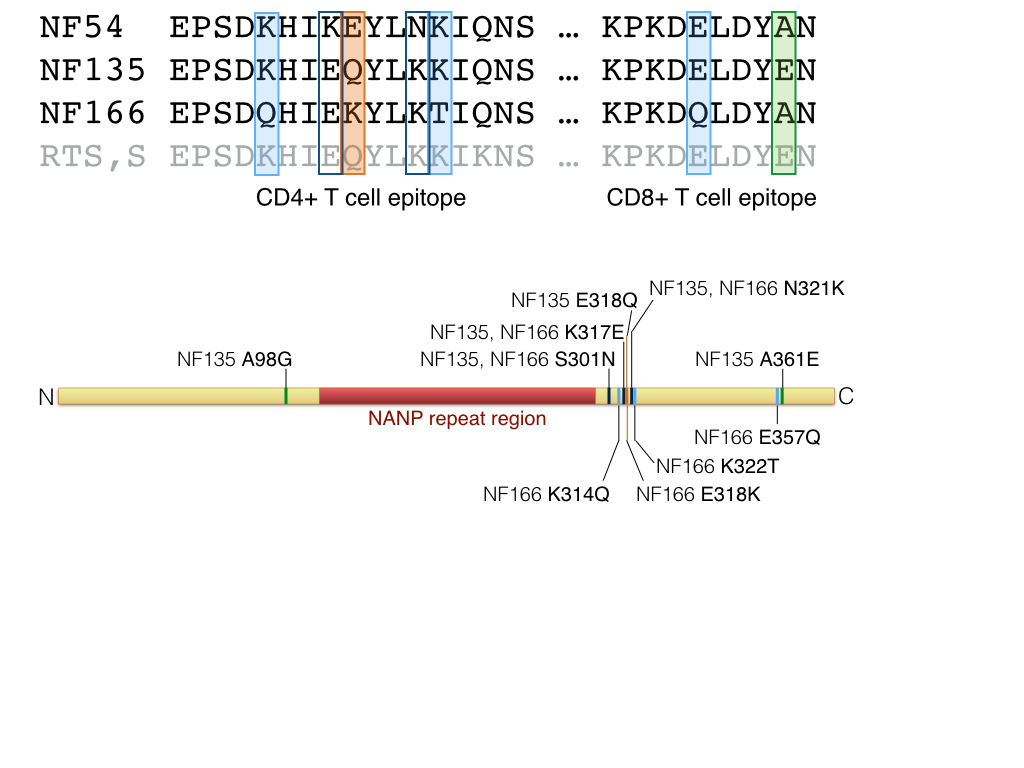


**Table S1: Whole-genome sequencing statistics.**

| **Strain** | **Mio read pairs** | **Median coverage (excl. apicoplast and mitochondrial DNA)** | **% genome with min. 5x coverage** | **SNPs** | **Indels** |
| --- | --- | --- | --- | --- | --- |
| NF54 | 3.5 | 20 | 94.7 | — | — |
| NF135.C10 | 4.8 | 25 | 95.0 | 13,352 | 3863 |
| NF166.C8 | 5.6 | 30 | 95.6 | 12,418 | 3566 |

**Table S2: Mosquito salivary gland infectivity and sporozoite load of the three clones.**Mean mosquito salivary gland infectivity and sporozoite load determined one day prior to challenge infection by dissecting a sample of 10 mosquitoes per strain.

|  | **No. volunteers** | **Sporozoite load** | **Percent infectivity** |
| --- | --- | --- | --- |
| *Cohort 1* |  |  |  |
| **NF54** | 4 | 26,500 | 90 |
| **NF135.C10** | 8 | 18,000 | 100 |
| **NF166.C8** | 6 | 59,500 | 100 |
| *Cohort 2* |  |  |  |
| **NF54** | 6 | 44,300 | 100 |
| **NF135.C10** | 7 | 23,500 | 80 |
| **NF166.C8** | 8 | 17,000 | 90 |
